# A Comparison of Cost Effectiveness Using Data from Randomized Trials or Actual Clinical Practice: Selective Cox-2 Inhibitors as an Example

**DOI:** 10.1371/journal.pmed.1000194

**Published:** 2009-12-08

**Authors:** Tjeerd-Pieter van Staa, Hubert G. Leufkens, Bill Zhang, Liam Smeeth

**Affiliations:** 1 General Practice Research Database, London, United Kingdom; 2 Utrecht Institute for Pharmaceutical Sciences, Utrecht University, Utrecht, The Netherlands; 3 London School of Hygiene & Tropical Medicine, London, United Kingdom; University of Bern, Switzerland

## Abstract

**Background:**

Data on absolute risks of outcomes and patterns of drug use in cost-effectiveness analyses are often based on randomised clinical trials (RCTs). The objective of this study was to evaluate the external validity of published cost-effectiveness studies by comparing the data used in these studies (typically based on RCTs) to observational data from actual clinical practice. Selective Cox-2 inhibitors (coxibs) were used as an example.

**Methods and Findings:**

The UK General Practice Research Database (GPRD) was used to estimate the exposure characteristics and individual probabilities of upper gastrointestinal (GI) events during current exposure to nonsteroidal anti-inflammatory drugs (NSAIDs) or coxibs. A basic cost-effectiveness model was developed evaluating two alternative strategies: prescription of a conventional NSAID or coxib. Outcomes included upper GI events as recorded in GPRD and hospitalisation for upper GI events recorded in the national registry of hospitalisations (Hospital Episode Statistics) linked to GPRD. Prescription costs were based on the prescribed number of tables as recorded in GPRD and the 2006 cost data from the British National Formulary. The study population included over 1 million patients prescribed conventional NSAIDs or coxibs. Only a minority of patients used the drugs long-term and daily (34.5% of conventional NSAIDs and 44.2% of coxibs), whereas coxib RCTs required daily use for at least 6–9 months. The mean cost of preventing one upper GI event as recorded in GPRD was US$104k (ranging from US$64k with long-term daily use to US$182k with intermittent use) and US$298k for hospitalizations. The mean costs (for GPRD events) over calendar time were US$58k during 1990–1993 and US$174k during 2002–2005. Using RCT data rather than GPRD data for event probabilities, the mean cost was US$16k with the VIGOR RCT and US$20k with the CLASS RCT.

**Conclusions:**

The published cost-effectiveness analyses of coxibs lacked external validity, did not represent patients in actual clinical practice, and should not have been used to inform prescribing policies. External validity should be an explicit requirement for cost-effectiveness analyses.

*Please see later in the article for the Editors' Summary*

## Abstract 2

Tjeerd-Pieter van Staa and colleagues estimate the likely cost effectiveness of selective Cox-2 inhibitors prescribed during routine clinical practice, as compared to the cost effectiveness predicted from randomized controlled trial data.

## Editors' Summary

**Background:**

Before a new treatment for a specific disease becomes an established part of clinical practice, it goes through a long process of development and clinical testing. This process starts with extensive studies of the new treatment in the laboratory and in animals and then moves into clinical trials. The most important of these trials are randomized controlled trials (RCTs), studies in which the efficacy and safety of the new drug and an established drug are compared by giving the two drugs to randomized groups of patients with the disease. The final hurdle that a drug or any other healthcare technology often has to jump before being adopted for widespread clinical use is a health technology assessment, which aims to provide policymakers, clinicians, and patients with information about the balance between the clinical and financial costs of the drug and its benefits (its cost-effectiveness). In England and Wales, for example, the National Institute for Health and Clinical Excellence (NICE), which promotes clinical excellence and the effective use of resources within the National Health Service, routinely commissions such assessments.

**Why Was This Study Done?:**

Data on the risks of various outcomes associated with a new treatment are needed for cost-effectiveness analyses. These data are usually obtained from RCTs, but although RCTs are the best way of determining a drug's potency in experienced hands under ideal conditions (its efficacy), they may not be a good way to determine a drug's success in an average clinical setting (its effectiveness). In this study, the researchers compare the data from RCTs that have been used in several published cost-effectiveness analyses of a class of drugs called selective cyclooxygenase-2 inhibitors (“coxibs”) with observational data from actual clinical practice. They then ask whether the published cost-effectiveness studies, which generally used RCT data, should have been used to inform coxib prescribing policies. Coxibs are nonsteroidal anti-inflammatory drugs (NSAIDs) that were developed in the 1990s to treat arthritis and other chronic inflammatory conditions. Conventional NSAIDs can cause gastric ulcers and bleeding from the gut (upper gastrointestinal events) if taken for a long time. The use of coxibs avoids this problem.

**What Did the Researchers Do and Find?:**

The researchers extracted data on the real-life use of conventional NSAIDs and coxibs and on the incidence of upper gastrointestinal events from the UK General Practice Research Database (GPRD) and from the national registry of hospitalizations. Only a minority of the million patients who were prescribed conventional NSAIDs (average cost per prescription US$17.80) or coxibs (average cost per prescription US$47.04) for a variety of inflammatory conditions took them on a long-term daily basis, whereas in the RCTs of coxibs, patients with a few carefully defined conditions took NSAIDs daily for at least 6–9 months. The researchers then developed a cost-effectiveness model to evaluate the costs of the alternative strategies of prescribing a conventional NSAID or a coxib. The mean additional cost of preventing one gastrointestinal event recorded in the GPRD by using a coxib instead of a NSAID, they report, was US$104,000; the mean cost of preventing one hospitalization for such an event was US$298,000. By contrast, the mean cost of preventing one gastrointestinal event by using a coxib instead of a NSAID calculated from data obtained in RCTs was about US$20,000.

**What Do These Findings Mean?:**

These findings suggest that the published cost-effectiveness analyses of coxibs greatly underestimate the cost of preventing gastrointestinal events by replacing prescriptions of conventional NSAIDs with prescriptions of coxibs. That is, if data from actual clinical practice had been used in cost-effectiveness analyses rather than data from RCTs, the conclusions of the published cost-effectiveness analyses of coxibs would have been radically different and may have led to different prescribing guidelines for this class of drug. More generally, these findings provide a good illustration of how important it is to ensure that cost-effectiveness analyses have “external” validity by using realistic estimates for event rates and costs rather than relying on data from RCTs that do not always reflect the real-world situation. The researchers suggest, therefore, that health technology assessments should move from evaluating cost-efficacy in ideal populations with ideal interventions to evaluating cost-effectiveness in real populations with real interventions.

**Additional Information:**

Please access these Web sites via the online version of this summary at http://dx.doi.org/10.1371/journal.pmed.1000194.

- The UK National Institute for Health Research provides information about health technology assessment
- The National Institute for Health and Clinical Excellence Web site describes how this organization provides guidance on promoting good health within the England and Wales National Health Service
- Information on the UK General Practice Research Database is available
- Wikipedia has pages on health technology assessment and on selective cyclooxygenase-2 inhibitors (note that Wikipedia is a free online encyclopedia that anyone can edit; available in several languages)

## Introduction

Many countries require health technology assessments when deciding on adopting new healthcare technologies. Recently, the American College of Physicians recommended the establishment of an organization for the generation and review of cost-effectiveness analyses [1]. In England and Wales, formal cost-effectiveness analyses are now required and several years ago the National Institute for Health and Clinical Excellence (NICE) was established to balance the financial costs and clinical benefits of health technologies and evaluate their cost effectiveness [2],[3]. It would be of interest to evaluate the experience in England and Wales and evaluate whether previous cost-effectiveness analyses adequately informed and guided medical practice.

Selective cyclooxygenase-2 inhibitors (coxibs) ranked, before September 2004, among the most commonly used medications in the world. They were developed to minimize the upper gastrointestinal (GI) side-effects of conventional nonsteroidal anti-inflammatory drugs (NSAIDs). There have been at least 33 published studies that evaluated the cost effectiveness of coxibs (celecoxib, rofecoxib, etoricoxib, or lumiracoxib) relative to that of conventional NSAIDs [4]–[36]. Although the use of coxibs has now changed following the findings of cardiovascular harm [37], they provide a good example of a drug with recently published cost-effectiveness analyses that were used to inform prescribing policies [8],[36]. Randomised clinical trial (RCT) data were used for the estimates of the rates of the upper GI events in all cost-effectiveness studies, except those conducted prior to the completion of large RCTs [4]–[7]. RCT data are still widely used not only for efficacy estimates but also for costs and incidence estimates [38]–[40]. While RCTs undoubtedly provide the best evidence for efficacy, they may not be the best source of costing data [41]. In addition, it is unclear whether RCT estimates on the incidence of outcomes represent the experience of patients in actual clinical practice [42]. However, there has been little empirical investigation of these issues. The objective of this study was to evaluate the external validity of published cost-effectiveness studies by comparing the data used in these studies to observational data from actual clinical practice and whether these studies should have been used to inform prescribing policies. Coxibs were used as an example.

## Methods

### Design of the Cost-Effectiveness Model

A basic cost-effectiveness model was developed evaluating two alternative strategies: prescription of a conventional NSAID or coxib. The model estimated the incremental cost of preventing one upper GI event with coxibs in a large representative UK population that had been prescribed anti-inflammatory medication during 1990–2006 for any medical condition. The prescriptions costs and the number of cases with upper GI events during current exposure to coxibs were compared in a simulation model to those with conventional NSAIDs.

### Risks of Upper GI Events

The upper GI events included clinically symptomatic gastroduodenal ulcers and complications such as upper GI hemorrhage. Two data sources were used to estimate the risks of upper GI events. Firstly, data were derived from existing RCTs. All published cost-effectiveness analyses conducted since 2000 used RCT data for the estimates of the risks of upper GI events [8]–[36]. Literature was searched for large RCTs (including over 2,000 patients) or meta-analyses of RCTs with prevention of upper GI events as primary outcome. A total of 11 large RCTs or meta-analyses was identified [43]–[53]. Secondly, data from actual clinical practice were used to estimate the absolute risk of upper GI events among patients using NSAIDs and coxibs. All patients aged 40 y or older prescribed conventional NSAIDs or coxibs and registered in the General Practice Research Database (GPRD) were identified. The GPRD comprises the anonymized computerized medical records of general practitioners (GPs). GPs play a key role in the UK health care system, as they are responsible for primary health care and specialist referrals. Patients are affiliated to a practice, which centralizes the medical information from the GPs, specialist referrals, and hospitalizations. The data recorded in the GPRD include demographic information, prescription details, clinical events, preventive care provided, specialist referrals, and hospital admissions and their major outcomes [54]. GPRD data collection started in 1987 and currently includes data on over 10 million patients. Two outcomes were measured and considered separately in the analyses. The first outcome concerned a GPRD record of upper GI events (as based on a GP diagnosis or based on a hospital or consultant letter as recorded into GPRD by the GP). The second outcome concerned hospitalizations for upper GI events, as obtained from the national registry of hospital admissions in England (Hospital Episode Statistics). Each hospital records the dates of admission and discharge and diagnoses of all hospitalizations (data from 2001 to 2006 were used). These hospital data can now be linked individually and anonymously to patients in English GPRD practices. The hospitalizations for upper GI events included the ICD-10 codes for gastric, duodenal, peptic, or gastrojejunal ulcer and gastritis or duodenitis (K25–K29).

The GPRD study population was followed from the first NSAID prescription to the patient's death, patient's transfer out of the general practice, or the last GPRD data collection available for this study (first quarter of 2006), whichever date came first. The follow-up of the study population was divided into periods of current and past exposure, with patients moving between these exposures. Current exposure was the time-period starting at the date of a prescription up to 3 mo after the end of the prescription duration. On average, prescriptions for conventional NSAIDs and coxibs provided for a treatment of 28 d. Past exposure was the remaining time of the follow-up period of a patient (i.e., the time distant from a prescription). In this population, the incidence rates of upper GI events (i.e., the number of cases per 1,000 person-years) were estimated during current and past exposure overall and by age, gender, exposure characteristics, and GI risk factors. Poisson regression was used to estimate the relative risk (RR) of upper GI events during current compared to past exposure. All these analyses were done separately for conventional NSAIDs and coxibs. In the analysis of conventional NSAIDs, patients were censored at the first coxib prescription.

### Exposure Characteristics

The published cost-effectiveness studies estimated the cost effectiveness for daily treatment for continuous periods of time [4]–[36]. The large RCTs all evaluated long-term NSAID exposure (ranging from 3 mo to 3 y) in patients with either rheumatoid arthritis (RA) or osteoarthritis (OA), requiring chronic or continuous NSAID therapy for the duration of the trial.

The longitudinal prescription histories in GPRD were used to determine the exposure characteristics (daily or intermittent and short- or long-term use). The medication possession ratio (i.e., the proportion of time covered by medication use) was estimated for each NSAID prescription that had a prior prescription in the 6 mo before. The medication possession ratio was the expected duration of NSAID exposure of the previous prescription divided by the time from between these two prescriptions. Prescriptions that were issued at least 6 mo after the previous NSAID prescriptions were classified as exposure with long gaps.

First-time exposure was the first NSAID prescription issued at least 1 y after start of GPRD data collection. At each NSAID prescription, the number of NSAID prescribed in the 1 y before was also calculated approximating the prior exposure duration (short-term, ≤4; medium-term, 5–11; and long-term exposure, ≥11 prior prescriptions). Prescriptions with missing information on the expected duration of use were classified into a separate category.

In the UK, ibuprofen is available over the counter without prescription. Patients need to pay a charge for GP prescriptions, except elderly and patients with low incomes. Further details on the prescribing patterns of conventional NSAIDs and coxibs can be found elsewhere [55],[56].

### Risk Factors for Upper GI Events

In the GPRD population, the GI risk factors were estimated at each prescription, including age of 65 y or older, recent prescribing in the 6 mo before of oral glucocorticoids, or anticoagulants, and a history of peptic upper GI bleeding, ischemic heart disease, hypertension, heart, renal or liver failure, or diabetes mellitus. These risk factors were included in NSAID prescribing guidelines from NICE [57]. Additional upper GI risk factors measured in this study included calendar year, the number of visits to the GP in the 6 to 12 mo before, smoking history and use of alcohol and body mass index (where available), medical history of OA or RA, and concomitant prescribing of aspirin or gastro-protective (ulcer-healing) drugs (British National Formulary 1.3).

### Clinical Effects of Coxibs

In order to derive an estimate of the beneficial effects of coxibs on the risk of upper GI events, a meta-analysis of 11 RCTs was used. This meta-analysis reported a relative risk reduction (RRR) of 51% of clinically symptomatic ulcers with coxibs (RR of 0.49; 95% confidence interval [CI] 0.38–0.62) [58]. We assumed in the simulation model that the risk of upper GI events, as observed in GPRD in users of conventional NSAIDs, would have been reduced by 51% if a coxib had been prescribed. Conversely, we assumed that the risk during current coxib exposure in GPRD would have increased by 51%, if a conventional NSAID had been prescribed. In the main analysis, it was assumed that the risk reduction due to coxibs would start immediately, similar to the assumptions in the published cost-effectiveness studies [4]–[36]. As several RCTs reported an onset of coxib effect only 1 to 6 mo after starting exposure [45],[46],[52],[53] (i.e., diverging of the risks between the coxib and control groups), a sensitivity analysis was conducted assuming a delayed onset of effect (after 1 or 6 mo).

### Prescription Costs

Prescription costs of each NSAID and coxib prescription in GPRD were estimated using the prescribed number of tables and the 2006 cost data from the British National Formulary. The cost data were converted from British pounds into US dollars using an exchange of £1 to US$2 (approximately the exchange rate at the end of 2006). As prescription costs varied substantially and the use of a single cost difference would be incorrect, the prescriptions of conventional NSAIDs and coxibs were ranked by costs and the incremental cost was based on the cost difference at each rank between conventional NSAIDs and coxibs. In a sensitivity analysis, the cost estimates from a recent UK assessment report were used (US$5.60 per month for a conventional NSAID and US$41.28 for a coxib) [31].

### Simulation Model

Simulation methodology was used to estimate the incremental cost of preventing one upper GI event during current exposure to coxibs. The number of upper GI cases avoided by coxibs was based on the RRR of the drug effect and the patient-specific incidence of upper GI events as estimated in the Poisson regression. The random variability was determined as follows. The event probabilities were randomly selected from a normal distribution on the basis of its mean and standard deviation. The coxib RRR used in each simulation was randomly selected from a normal distribution based on the RRR and 95% CI reported in literature [58]. The simulation was repeated 250 times and nonparametric bootstrapping techniques were then used to estimate the 95% CIs (i.e., the 2.5% and 97.5% percentiles) [59].

## Results

Table 1 shows the rate of upper GI events in the large RCTs of coxibs. Study patients were restricted to those who required long-term NSAID exposure and the indication for treatment was mostly OA or RA. Both the CLASS and VIGOR studies did not apply “intention to treat” statistical analyses, but restricted the analyses to events that occurred during treatment or within 14 d of discontinuation of treatment.

**Table 1:** Characteristics of patients and NSAID exposure in the large coxib RCTs or meta-analyses and in actual clinical practice (GPRD).

Study	*n*	Indication	Extent of NSAID Exposure in RCT	Daily Dose Coxibs	Rate of Upper GI Events^a^	
					Conventional NSAIDs	Coxibs
**Langman meta-analysis 1999 ** [**44**]	**5,435**	RA or OA	Not reported	12.5–50 mg rofecoxib	26.0	13.3
**VIGOR 2000 ** [**45**]	**8,076**	RA	Daily for 11 mo	50 mg rofecoxib	45.0	21.0
**CLASS 2000 ** [**46**]	**8,059**	RA or OA	Daily for 6 mo	800 mg celecoxib	36.8	22.2
**Goldstein meta-analysis 2000 ** [**47**]	**9,144**	RA or OA	Not reported	50–800 mg celecoxib	16.8	2.0
**ADVANTAGE 2003 ** [**48**]	**5,557**	OA	Daily for 12 wk	25 mg rofecoxib	14.3	3.1
**Watson meta-analysis 2004 ** [**49**]	**17,072**	RA or OA	Not reported	12.5–50 mg rofecoxib	18.7	7.4
**TARGET 2004 ** [**50**]	**18,325**	OA	Daily for 1 y	400 mg lumiracoxib	9.1	3.2
**SUCCESS 2006 ** [**51**]	**13,274**	OA	Daily for 3 mo	200 or 400 mg celecoxib	21.1	10.0
**MEDAL 2007 ** [**52**]	**34,701**	RA or OA	Daily for 3 y	60 or 90 mg etoricoxib	9.7	6.7
**Ramey meta-analysis ** [**53**]	**5,441**	RA, OA, or ankylosing spondylitis	Not reported	60–120 mg etoricoxib	24.7	10.0
**Actual clinical practice (GPRD)**	**>1 million**	Heterogeneous	Variable	Variable	6.0 (GP recorded)	5.9 (GP recorded)
	—	—	—	—	3.8 (Hospitalization)	5.3. (Hospitalization)

a Number of cases per 1,000 person-years.

The GPRD study population included 971,426 patients prescribed conventional NSAIDs and 148,592 prescribed coxibs. A medical history of RA or OA was present in 23.0% of the conventional NSAID users and 45.9% of the coxib users. They were given a total of 8.5 million conventional NSAID prescriptions and 0.9 million coxib prescriptions. The longitudinal prescription histories indicated that a large proportion of patients used the NSAIDs intermittently. Only about 34.5% of conventional NSAID and 44.2% of coxib prescriptions were given to patients with enough medication for longer term daily exposure (Table 2). The RRs of upper GI events during current exposure (compared to past exposure) were higher in those with continuous NSAID exposure and lower with incidental exposure. As shown in Table 3, the rate of upper GI events (as recorded by the GP) and of upper GI hospitalizations during current exposure to conventional NSAIDs decreased over calendar time by 5%–8% per year (*p*-value for tests of linear trend <0.0001 and 0.04, respectively). The rate of upper GI hospitalizations during current exposure to conventional NSAID users in GPRD was 12-fold lower than the rate reported in the VIGOR RCT (3.8 and 45.0 per 1,000 person-years, respectively).

**Table 2:** Distribution of exposure characteristics of conventional NSAIDs and coxibs and RRs of upper GI events during current exposure (compared to past exposure).

Exposure Characteristics			Percent of Rx	Mean Age	Women (%)	GI Risk Factors (%)	OA or RA (%)	Repeat NSAID Rx within 3 mo	Crude RR of GP Recorded Upper GI Events (95% CI)	Crude RR of Hospitalization for Upper GI Events (95% CI)
**Conventional NSAIDs**										
**First-time**			8.3%	57.8	52.9%	44.7%	9.5%	24.7%	1.9 (1.8–2.1)	1.6 (1.3–2.1)
**Long gap**			19.4%	60.9	57.6%	54.7%	24.6%	29.1%	1.5 (1.4–1.6)	1.2 (1.0–1.5)
**Medication**	**Very low**		11.7%	64.3	59.6%	67.3%	39.1%	61.7%	1.9 (1.7–2.0)	1.9 (1.5–2.4)
**Possession**	**Low**		9.7%	65.7	59.5%	72.1%	44.9%	78.8%	2.2 (2.0–2.4)	2.7 (2.1–3.4)
**Ratio**	**Moderate**		8.8%	65.9	60.2%	72.4%	47.5%	85.7%	2.6 (2.4–2.8)	3.0 (2.3–3.9)
	**High**	Short-term use	7.7%	65.0	59.6%	68.6%	36.2%	71.7%	3.5 (3.2–3.7)	2.7 (2.0–3.6)
		Medium-term	16.5%	67.2	61.2%	77.8%	51.0%	92.1%	3.5 (3.3–3.7)	3.2 (2.6–4.0)
		Long-term	18.0%	68.5	63.1%	82.5%	54.9%	98.1%	4.0 (3.7–4.2)	4.8 (4.0–5.9)
**Coxibs**										
**First-time**			2.7%	66.0	59.4%	69.7%	22.2%	39.7%	1.9 (1.3–2.7)	2.8 (1.8–4.5)
**Long gap**			10.1%	66.8	66.9%	73.5%	42.8%	44.4%	1.6 (1.3–1.9)	1.4 (1.0–2.0)
**Medication**	**Very low**		8.5%	66.3	67.9%	73.4%	53.2%	65.4%	1.3 (1.0–1.7)	1.4 (0.9–2.1)
**Possession**	**Low**		13.2%	67.3	67.0%	77.9%	57.7%	84.2%	1.6 (1.3–2.0)	1.2 (0.8–1.9)
**Ratio**	**Moderate**		9.9%	67.3	68.2%	77.4%	58.0%	87.5%	1.7 (1.3–2.3)	1.0 (0.601.7)
	**High**	Short-term use	11.5%	68.7	67.7%	78.8%	47.2%	78.9%	2.4 (1.9–2.9)	2.4 (1.7–3.3)
		Medium-term	19.2%	68.8	68.7%	81.6%	59.2%	92.6%	2.7 (2.2–3.2)	2.2 (1.7–3.0)
		Long-term	25.0%	70.3	70.0%	86.0%	63.9%	97.3%	2.4 (2.0–2.9)	3.1 (2.4–4.0)

Each NSAID prescription was classified according to first-ever use, long gap (previous prescription at least 6 mo before), and short gap (previous prescription within the last 6 mo). The medication possession ratio was estimated for the prescriptions issued after a short gap and divided into very low (<0.40), low (0.40–0.59), moderate (0.60–0.79), and high (0.80+). Short-term use was defined ≤4 prescriptions in the 1 y before, medium-term 5–11, and long-term ≥11 prior NSAID prescriptions. Rx, prescription.

**Table 3:** The incidence rate of upper GI events during current exposure to conventional NSAIDs or coxibs stratified by number of risk factors and calendar time.

Risk Factor		Percent of Rx	GP Recorded Upper GI Events		Hospitalization for Upper GI Events^a^	
			No Cases	Rate^b^	No Cases	Rate^b^
**Conventional NSAIDs**	—	—	—	—	—	—
**Calendar year**	**1990–1993**	17.5%	2,432	10.2	—	—
	**1994–1997**	23.4%	2,413	7.6	—	—
	**1998–2001**	31.8%	1,940	4.5	172	4.4
	**2002–2005**	27.4%	1,414	3.7	664	3.7
**Number of major upper GI risk factors**	**0**	31.1%	1,334	2.5	93	1.1
	**1**	31.1%	2,338	5.5	192	2.9
	**2+**	37.8%	4,742	10.5	571	7.7
**Coxibs**	**—**	—	—	—	—	—
**Calendar year**	**2000–2001**	13.2%	138	7.0	29	6.0
	**2002–2005**	86.8%	600	5.7	249	5.3
**Number of major upper GI risk factors**	**0**	20.8%	64	2.1	15	1.2
	**1**	29.3%	146	3.9	51	3.3
	**2+**	49.7%	537	9.1	214	8.6

a Hospitalization data were derived from a subset of GPRD practices and covered the time period from 2001 to 2006.

b Rate was the number of cases per 1,000 person-years.

Rx, prescription.

**Figure 1:**
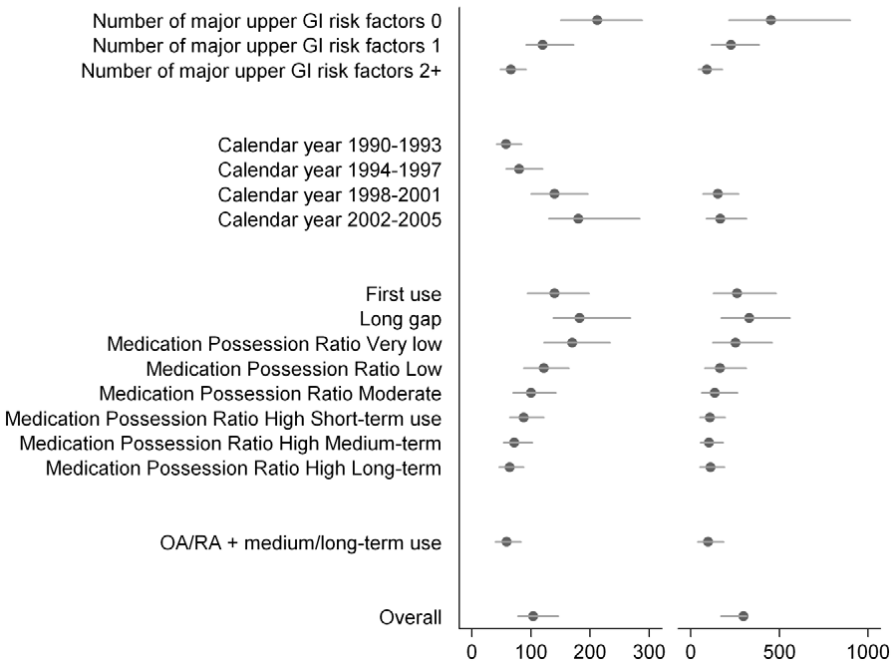
The mean cost in US$ per case avoided with coxibs (and 95% CI) overall and stratified by the number of major risk factors, calendar year, and exposure characteristics. Middle panel, GP recorded upper GI events; right panel, hospitalization for upper GI events. The exposure characteristics of each NSAID prescription was classified according to first-ever use, long gap (previous prescription at least 6 mo before), and short gap (previous prescription within the last 6 mo). The medication possession ratio was estimated for the prescriptions issued after a short gap and divided into very low (<0.40), low (0.40–0.59), moderate (0.60–0.79), and high (0.80+). Short-term use was defined as ≤4 prescriptions in the 1 y before, medium-term 5–11, and long-term ≥11 prior NSAID prescriptions. *x*-Axis, mean cost in US$ per case avoided; *y*-axis: population subgroup.

The mean cost of a conventional NSAID prescription was US$17.80 (range of US$4.56 at 5th percentile to US$47.36 at 95th percentile). For coxibs, the mean cost was US$47.04 (range from US$18.62 to US$83.96). The mean incremental cost of replacing a conventional NSAID with a coxib was US$29.24. The mean cost of preventing one clinical upper GI event by substituting the conventional NSAID by a coxib was US$104k (95% CI US$74–146k) using GPRD estimates for the risk of upper GI events (Table 4). The cost effectiveness varied substantially by calendar year and exposure characteristics (Figure 1). As shown in Table 4, there was a large heterogeneity across the study population in the costs of preventing one upper GI event. In patients with two or more upper GI risk factors, 71.9% of the prescriptions had a cost below US$100k per case avoided in long-term users while 36.6% in intermittent users (with long gaps).

**Table 4:** The heterogeneity in the cost per case avoided with coxibs stratified by the number of major risk factors and exposure characteristics (with the cost per case avoided estimated for each individual prescription).

Exposure Characteristics			GP Recorded Upper GI Events								
			No Major Upper GI Risk Factor			One Major Upper GI Risk Factor			Two+ Major Upper GI Risk Factors		
			Percent of Rx below 100k	Percent of Rx 100–200k	Percent of Rx 200+k	Percent of Rx below 100k	Percent of Rx 100–200k	Percent of Rx 200+k	Percent of Rx below 100k	Percent of Rx 100–200k	Percent of Rx 200+k
**First-time**			11.1	22.0	66.8	31.0	28.1	40.9	51.7	26.0	22.3
**Long gap**			5.9	15.5	78.6	17.4	25.3	57.3	36.6	29.1	34.3
**Medication**	**Very low**		5.7	18.3	76.0	16.0	25.2	58.8	31.0	26.4	42.6
**Possession**	**Low**		8.9	25.0	66.1	23.7	31.3	45.0	44.0	30.6	25.4
**Ratio**	**Moderate**		12.9	30.4	56.7	31.8	32.7	35.5	52.6	28.7	18.8
	**High**	Short-term use	16.8	34.5	48.7	33.0	36.6	30.4	56.9	29.5	13.6
		Medium-term	23.1	35.0	41.9	43.1	33.9	23.0	64.9	24.7	10.4
		Long-term	28.4	35.5	36.1	53.2	29.0	17.8	71.9	20.5	7.6

# Each NSAID prescription was classified according to first-ever use, long gap (previous prescription at least 6 mo before), and short gap (previous prescription within the last 6 mo). The medication possession ratio was estimated for the prescriptions issued after a short gap and divided into very low (<0.40), low (0.40–0.59), moderate (0.60–0.79), and high (0.80+). Short-term use was defined ≤4 prescriptions in the 1 y before, medium-term 5–11, and long-term ≥11 prior NSAID prescriptions. Rx, prescription.

The cost-effectiveness estimates worsened with a delayed coxib effect (Table 5). Conversely, the cost effectiveness of coxibs improved substantially when using RCT data for the risk of upper GI events (the mean cost was US$20k using the CLASS RCT [46] and US$16k using the VIGOR RCT [45]).

**Table 5:** Sensitivity analyses of the population mean of the cost per case avoided with coxibs using different assumptions for onset of coxib effect and event probabilities.

Model Assumptions	Mean Cost in US$ per Case Avoided (95% CI)	Percent of Rx below US$20k	Percent of Rx below US$100k
**Event probabilities based on rates observed in GPRD conventional NSAID users**			
**Overall**	104k (78–146k)	2.4	30.4
**Onset of coxib upper GI effects: 1 month**	212k (156–296k)	1.1	14.8
**6 mo**	>1 million	0	0
**Coxib effect only in patients with long-term use with high medication possession ratio (as studied in RCTs) and no effect in other patients**	310k (222–430k)	1.1	10.7
**Prescription costs based on UK assessment report ** [**31**]	144k (108–208k)	0.7	18.4
**Event probabilities based on rates observed in GPRD coxib users**	120k (56–208k)	2.6	31.3
**Event probabilities based on rates observed in conventional NSAID users in RCTs**			
**VIGOR RCT ** [**45**]	16k (12–20k)	77.9	99.0
**CLASS RCT ** [**46**]	20k (16–26k)	57.7	98.8
**Rofecoxib meta-analysis ** [**44**]	28k (22–38k)	16.5	98.0
**Celecoxib meta-analysis ** [**47**]	42k (34–54k)	5.9	96.3
**Etoricoxib meta-analysis ** [**53**]	28k (24–40k)	14.7	97.9

Rx, prescription.

## Discussion

Health technology assessments frequently use data from randomized trials for estimates of absolute risks of events and patterns of drug use. Using coxibs as an example, we have shown that cost-effectiveness analyses produced markedly different results depending on the source of the data used in the modeling. The cost effectiveness of coxibs was far worse when the analyses were based on data from actual clinical practice rather than RCTs. The use of data from actual clinical practice rather than RCTs would have radically altered the conclusions of health technology appraisals of coxibs.

There are several reasons for the substantive differences in results using actual clinical practice or RCT data. The incidence of upper GI events was lower among patients in GPRD compared to those in RCTs. In GPRD, there was an almost 3-fold reduction over calendar time in the incidence of upper GI events. This secular trend is consistent with that observed in Canada for the rate of hospital admission for upper GI events [60]. Furthermore, the cost-effectiveness analyses evaluated long-term daily use of coxibs in patients with RA or OA, while most patients in actual clinical practice did not have these conditions or used NSAIDs intermittently or for short periods of time. A further difference in the results of cost effectiveness may be related to the assumptions for prescription costs. Single estimates for costs were used in published cost-effectiveness models, while in daily practice there is a substantive variability in prescription costs for NSAIDs. Lastly, the published coxib cost-effectiveness studies described simple scenarios of drug exposure and event probabilities assuming uniformity in the population, while this study found a huge variability between patients in type of NSAID exposure, incidence of upper GI events, and prescription costs. In this study, a large proportion of the patients with a major upper GI risk factor, recommended to be treated with coxibs in the UK [57], had a cost per upper GI event avoided in excess of US$100k. The best strategy for targeting coxibs cost-effectively to heterogeneous populations has not yet been established. The use of coxibs has now changed following the findings of cardiovascular harm [37]. This study did not address the appropriate prescribing of coxib on the basis of our current understanding of these cardiovascular risks.

RCTs provide the best evidence for establishing the efficacy (relative effects) of a treatment and have high internal validity due to randomization and blinding. But randomization and blinding do not ensure that the absolute event probabilities and costs, as observed in a RCT, will represent those in actual clinical practice and that RCTs have external validity. The “real world” includes an incredible diversity and complexity [61], while the “world of RCTs” applies strict criteria for patient inclusion and for treatment exposure. RCTs often have an artificial design, with more tests conducted and increased patient monitoring. Also, patients may not comply with treatment instructions particularly well in the “real” world, increasing costs and decreasing the benefits. Thus, the absolute figures obtained from a RCT may very well deviate from and not represent the “real world.” On the other hand, observational studies may provide reasonably good estimates of absolute event probabilities and costs in patients in actual clinical practice, but have major limitations in attributing causality and estimating the relative effects of a drug treatment, principally owing to confounding. Rather than considering RCTs as the ideal evidence for all information, cost-effectiveness studies could use could use meta-analyses of RCT data for the drug effect estimates and observational data for the absolute event probabilities and costs [62]. In addition to providing a better context, this approach would also limit the possibility that the best RCT data are selected for the cost-effectiveness analyses [63]. An alternative and even better approach would be to use large pragmatic RCTs for cost-effectiveness models. Pragmatic RCTs are conducted with patients who represent the full spectrum of the population to which the treatment might be applied and with interventions that have real-life (rather than ideal) compliance [64].

Cost-effectiveness analyses that are intended to guide medical practice should consider the characteristics of all possible patient subgroups that may be provided with the new technology. As an example, the prevalence of risk factors, the incidence of upper GI events, and the exposure characteristics of conventional NSAID users in actual clinical practice could have been described prior to assessing the cost effectiveness of coxibs. Such an analysis would have noted the selective characteristics of the patients enrolled in the large coxib RCTs and differences in exposure characteristics. Few patients in GPRD used conventional NSAIDs in the manner as tested in the coxib RCTs (i.e., long-term use with higher daily doses). Patients may not require regular treatment, may not comply with dosage instructions, or persist with treatment. A second consideration for cost-effectiveness studies should be to evaluate the extent that RCT evidence can be generalized and extrapolated to each of these various patient subgroups that may be provided with the new technology in actual clinical practice. As an example, it would have been noted that most conventional NSAID users would not have been eligible for inclusion into the large coxib RCTs and that there is rather limited evidence for beneficial effects of coxibs with short-term or intermittent use (as done by most patients). While it may be impossible to conduct RCTs in patients who use a treatment intermittently or who comply less (because of the required sample size), the uncertainty in generalizing RCT efficacy estimates to populations more diverse in patient and treatment characteristics should be considered explicitly [65]. None of the 33 published coxib cost-effectiveness studies analysed the external validity of the assumptions used [4]–[36]. They did not provide any guidance on the prescribing of coxibs to the majority of patients using conventional NSAIDs in actual clinical practice (e.g., those with short-term or intermittent use). The field of health technology assessments should move from evaluating cost efficacy in ideal (hypothetical) populations with ideal interventions to cost effectiveness in real populations with pragmatic interventions.

One of the key limitations of this study was that the classification of upper GI events may have differed between RCTs and GPRD/Hospital Episode Statistics. In most of the large RCTs, all potential upper GI events were adjudicated in a standard manner. In the CLASS celecoxib RCT, only one-third of the potential cases were included in the analysis [46]. GPRD is based on information diagnosed and collected in actual clinical practice. This lack of case adjudication may have overestimated the rate of upper GI events in GPRD. On the other hand, there may have been under-diagnosis and/or under-recording in GPRD. However, clinically significant events are generally well recorded in GPRD, as documented by various validation studies [54]. Specifically, the validity of the diagnosis of upper GI bleeding in the GPRD records was assessed in a sample of 96 people with a diagnosis of upper GI bleeding recorded in their electronic records. Hospital records were reviewed and the diagnosis confirmed in 95 out of the sample of 96 patients [66].

In conclusion, the coxib cost-effectiveness studies lacked external validity and more realistic estimates for event rates and costs could have produced markedly different results, sufficient to have led to different prescribing guidelines. External validity should be an explicit requirement for cost-effectiveness analyses.

## Abbreviations

CI: confidence interval
coxib: cyclooxygenase-2 inhibitors inhibitor
GI: gastrointestinal
GP: general practitioner
GPRD: UK General Practice Research Database
NSAID: nonsteroidal anti-inflammatory drug
OA: osteoarthritis
RA: rheumatoid arthritis
RCT: randomised clinical trial
RR: relative risk
RRR: relative risk reduction
